# Evaluation of the efficacy of insecticide-treated scarves to protect children from the trachoma vector *Musca sorbens* (Diptera: Muscidae): A phase II randomised controlled trial in Oromia, Ethiopia

**DOI:** 10.1016/j.eclinm.2022.101487

**Published:** 2022-06-08

**Authors:** Ailie Robinson, Laura Reis de Oliveira Gomes, Oumer Shafi Abdurahman, Wondu Alemayehu, Gemeda Shuka, Ewunetu Melese, Meseret Guye, Demitu Legesse, Eden Elias, Kedir Temam, Korso Hirpo Koro, Dereje Adugna, Fikre Seife, Muluadam Abraham Aga, Virginia Sarah, Saba M. Lambert, Stephen L. Walker, Esmael Habtamu, Anthony W. Solomon, Anna Last, David Macleod, Matthew J. Burton, James G. Logan

**Affiliations:** a Department of Disease Control, London School of Hygiene and Tropical Medicine, Keppel Street, London, WC1E 7HT, UK; b The Fred Hollows Foundation, P.O. Box 6307, Addis Ababa, Ethiopia; c International Centre for Eye Health, Department of Clinical Research, London School of Hygiene and Tropical Medicine, Keppel Street, London, WC1E 7HT, UK; d Oromia Regional Health Bureau, Addis Ababa, Ethiopia; e The Ethiopian Federal Ministry of Health, Disease Prevention and Control Directorate, Addis Ababa, Ethiopia; f Global Partnerships Executive, The Fred Hollows Foundation, 12-15 Crawford Mews, York Street, London W1H1LX; g Department of Clinical Research, London School of Hygiene and Tropical Medicine, Keppel Street, London, WC1E 7HT, UK; h Department of Control of Neglected Tropical Diseases, World Health Organization, Avenue Appia 20, 1202 Genève, Switzerland; i Department of Infectious Disease Epidemiology, London School of Hygiene and Tropical Medicine, Keppel Street, London, WC1E 7HT, UK; j National Institute for Health Research Biomedical Research Centre for Ophthalmology, Moorfields Eye Hospital NHS Foundation Trust and UCL Institute of Ophthalmology, London, UK.

**Keywords:** Musca sorbens, Trachoma, Personal protection, Insecticide-treated clothing, Eyeseeking flies, Vector-borne disease

## Abstract

**Background:**

The eye-seeking fly *Musca sorbens* can act as a vector for ocular *Chlamydia trachomatis*, causing trachoma, yet there has been very little research on control measures. We investigated whether insect repellent products, specifically insecticide-treated clothing, could provide personal protection to the user from eye-seeking flies.

**Methods:**

We first conducted a series of phase I laboratory studies to inform our choice of field intervention. We then conducted a phase II randomised controlled trial testing the efficacy of permethrin-treated scarves (PTS) in reducing fly-face contact in Oromia, Ethiopia. Children aged 4-10 years in full health and with no known adverse reactions to permethrin or other insecticides were allocated to either arm using restricted randomisation. Intervention arm children wore Insect Shield® versatile wraps (as PTS) for 28 days. The primary outcomes, fly-eye, -nose and -mouth contact, were assessed on the first day (0/30/60/180 minutes), on day 7 and on day 28. All participants present per timepoint were included in analyses. This trial was registered with ClinicalTrials.gov (NCT03813069).

**Findings:**

Participants were recruited to the field trial between 29/10/2019 and 01/11/2019, 58 were randomised to test or control arm. More fly (-eye, -nose and -mouth) contacts were observed in the PTS arm at baseline. After adjusting for baseline contact rates, across all timepoints there was a 35% decrease in fly-eye contacts in the PTS relative to control arm (rate ratio [RR] 0.65, 95% CI 0.52-0.83). Similar cross-timepoint reductions were seen for fly-nose and fly-mouth contacts (RR 0.69, 95% CI 0.51-0.92 and RR 0.79, 95% CI 0.62-1.01, respectively). All children were included on day 0. Two in the control arm were absent on day 7, one left the study and four were excluded from analysis at day 28. No adverse events occurred in the trial.

**Interpretation:**

*Musca sorbens* flies are sufficiently repelled by PTS to reduce fly-eye contacts for the wearer, thus possibly reducing the risk of trachoma transmission. Permethrin-treated scarves may therefore an alternative to insecticide space spraying for protection from these flies.

**Funding:**

Wellcome Trust.

**Research in context**

**Evidence before the study**

Despite the role of eye-seeking *Musca sorbens* in the transmission of the blinding eye disease trachoma, there are scant evidence-based measures for their control. Insect repellents and insecticide-treated clothing can provide personal protection to the user, and many arthropod species are susceptible to their active ingredients. We searched MEDLINE, Global Health, Embase Classic and Embase with no restrictions on date or language, combining all terms and synonyms for *M. sorbens* with search terms related to insect repellents, insecticides and insecticide treated clothing. We retrieved 38 publications, none of these studies tested the use of insect repellents or insecticide-treated clothing against *M. sorbens*.

**Added value of this study**

*Musca sorbens* populations, like other species of filth fly, can increase exponentially given favourable breeding conditions. For this reason, fly control by population suppression, including insecticide spraying, may not be an optimal control tool. However, personal protection from fly-eye contacts may be useful and appropriate in the trachoma-endemic context. In our phase II study in Oromia, central Ethiopia, children aged 4-10 years who wore permethrin-treated headscarves experienced fewer fly-eye contacts relative to those in a control group given no intervention. To our knowledge, this is the first study of the use of insecticide-treated clothing against *M. sorbens*. Our findings support further large-scale studies of the use of permethrin-treated headwear against eye-seeking flies.

**Implications of all the available evidence**

Permethrin-treated clothing should be further tested in large-scale phase III trials with both epidemiological (trachoma) and entomological (fly-eye contact) outcomes. Such studies will inform whether the distribution of permethrin-treated clothing within communities can reduce fly-eye nuisance at scale and bolster trachoma elimination programmes.

Alt-text: Unlabelled box

## Introduction

Trachoma is a progressive eye disease. It is the most common infectious cause of blindness globally,^1^ and a neglected tropical disease, affecting some of the world's poorest and most marginalised populations. Infection of the eyelids with the bacterium *Chlamydia trachomatis* (Ct) initially causes inflammation, which over time can lead to scarring, entropion, and blindness.^2^ *Chlamydia trachomatis* is an intracellular bacterium, whose unusual lifecycle involves stages that are adapted for extracellular life, called elementary bodies (EB). These are shed by an infected person in ocular and nasal secretions. From here they are transmitted to another person mainly via close contact, or contamination of surfaces. Routes of transmission are, therefore, multiple, and their relative importance is presumed to vary by setting.

*Musca sorbens* is a synanthropic ‘filth’ fly which feeds on ocular discharge and from mucous membranes of humans.^3^ This eye-seeking habit can cause *M. sorbens* to become contaminated with Ct EBs, then transport them from one person to another, acting as a mechanical vector of disease.^4^ In any given setting, the contribution of *M. sorbens* to Ct transmission is likely to be dependent on the local fly population density, as well as possible local variation in fly behaviour, biology or ecology. Partly because of this variability, the contribution of *M. sorbens* to trachoma transmission is not fully characterised nor understood. Further, practical control methods for this disease vector are lacking, with space spraying with pyrethroid insecticides being the only evidence-based option.^5^, ^6^, ^7^ These flies are diurnal and, in Ethiopia, exophilic, so tools commonly used to control other vector species, for example insecticide-treated bednets or indoor residual spraying, are not useful.

Personal protection from the aggressive eye-seeking behaviour would both protect against disease transmission and alleviate distress. Insect repellents are used world-wide to prevent nuisance biting by both vector and non-vector arthropod species. Commercially available topical repellents are rarely used by people in low-income countries with endemic arthropod-borne diseases, because of availability, cost and the impracticality of a product that requires repeat application. In some regions, plants with repellent properties are used, either by burning leaves or laying out fresh foliage,^8^^,^^9^ including in central Ethiopia where leafy branches of the Pepper tree, *Schinus mole,* are laid down in houses to deter flies. An alternative method of protection could be the use of insecticide-treated clothing (ITC). Insecticide-treated clothing incorporates insecticides that have spatially repellent properties or are contact irritants, and may be a practical and contextually appropriate means to deter eye-seeking flies. While ITC has been shown to provide protection from malaria and leishmaniasis in some contexts,^10^^,^^11^ more studies are needed to improve the evidence base of ITC to control disease.^12^

We hypothesised that insect repellents may provide personal protection against the eye-seeking *M. sorbens*. We anticipated that in areas with high fly density, an immediate benefit of reduced fly-face contact may encourage uptake of this intervention. We first screened the use of the topical repellents DEET (N,N-diethyl-3-methylbenzamide), IR3535 (ethyl 3-[acetyl(butyl)amino]propanoate), Picaridin (butan-2-yl 2-(2-hydroxyethyl)piperidine-1-carboxylate) and delta-Undecalactone (6-hexyloxan-2-one; dUDL), as well as Craghopper and Insect shield ITC garments (both containing permethrin (3-phenoxyphenyl)methyl 3-(2,2-dichloroethenyl)-2,2-dimethylcyclopropane-1-carboxylate), against *M. sorbens* fly-skin contact in laboratory bioassays. The screening process led to our testing topical IR3535 (Jungle Formula Kids) and permethrin-treated scarves (PTS) in a small-scale laboratory-based trial, which in turn informed our choice of intervention for the field trial. We then conducted a randomised controlled trial in Oromia, Ethiopia, testing whether PTS could protect against fly-face (herein used to refer to -eye, -nose and -mouth) contact in children aged 4-10 years.

## Methods

### Study design and participants

We first conducted a series of phase I studies to select our repellent intervention. After designing arm-in-cage laboratory bioassays for testing repellent products against the eye- and skin-seeking *M. sorbens*, we screened a range of these for possible efficacy (supplementary material, phase I studies). This work led to a small-scale preliminary laboratory study in which six participants tested the topical repellent Jungle Formula Kids® (JFK; intended as a positive control), and permethrin-treated scarves (PTS; two concentrations tested), for protection from skin contact by *M. sorbens* (full methods are given in Appendix 1, ‘supplementary phase I studies: preliminary laboratory trial’).

**Figure 1:**
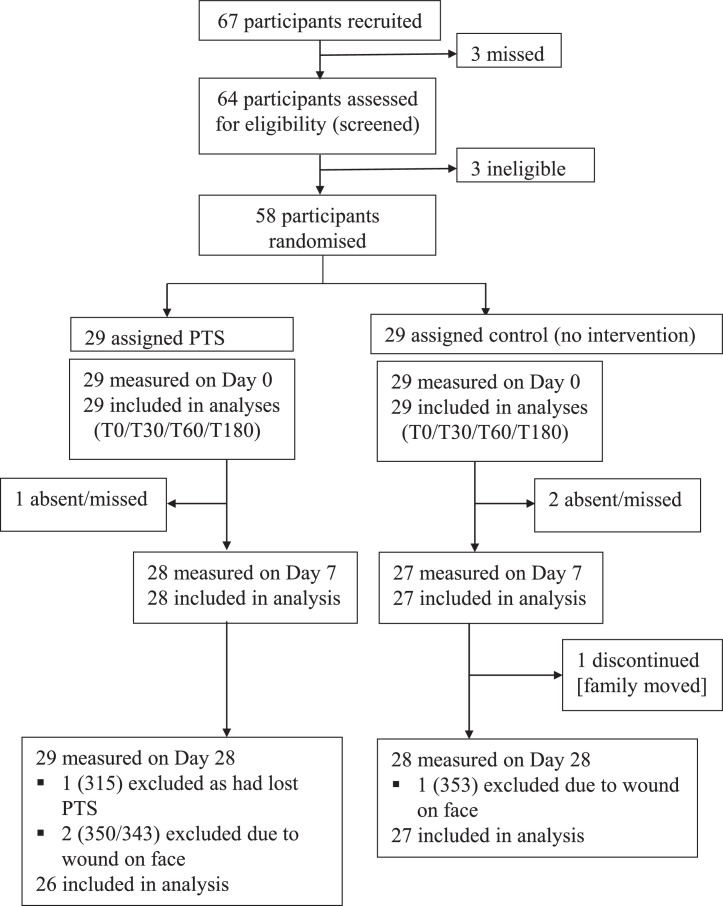
**Trial profile**. PTS=permethrin-treated scarf.

**Figure 2:**
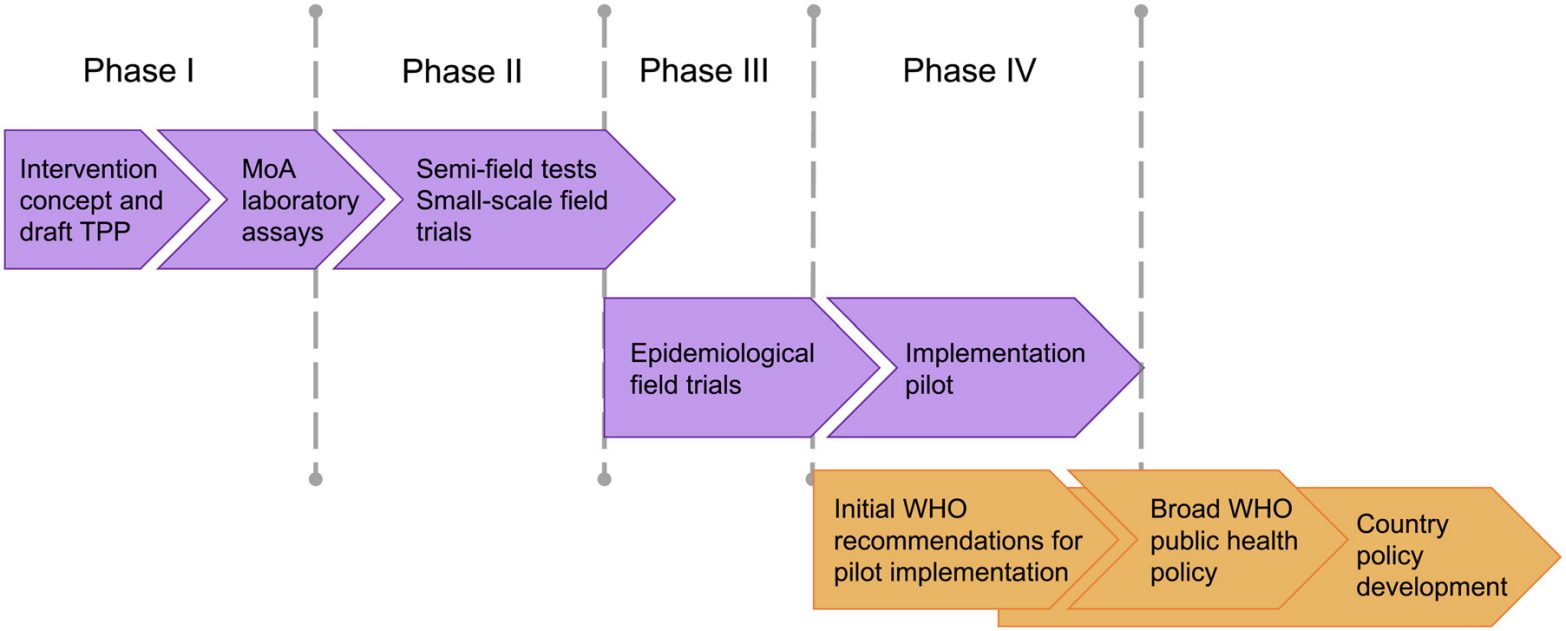
**Stages in development of a new vector control product**. The World Health Organization's (WHO) Vector Control Advisory Group categorises studies that test new vector control interventions into phases I-IV. In our phase I laboratory studies we designed a bioassay for testing repellent products against eye-seeking M. sorbens flies, screened a range of repellent products, then selected the field trial intervention product. Our randomised controlled field trial, testing the use of permethrin-treated scarves against eye-seeking flies, was a phase II study (entomological outcomes only). Phase III studies assess the efficacy of interventions against epidemiological outcomes and inform policymakers (image adapted from^13^; TPP, target product profile; MoA, mode of action).

The results of this preliminary study informed our choice of intervention for the subsequent phase II randomised controlled field trial (Figure 1, Figure 2), in which two parallel groups tested the protection afforded by permethrin-treated scarves (given to wear constantly), or a placebo scarf (only worn during the primary outcome observations), against fly-eye, -nose and -mouth contact. The field trial was conducted for 28 days in the Kubi Guta kebele of Shashemene woreda, Oromia, Ethiopia. This is a rural area with high levels of poverty and high fly density. Our previous work showed that the eye-seeking fly population is approximately 90% *M. sorbens*,^14^ and that fly densities are particularly high during the hot season, from approximately early November until late March. Community sensitisation was carried out prior to engagement with potential participants, in which our study was introduced and described to key stakeholders. During these conversations permission to conduct the study in the area was sought and granted. Eligibility criteria specified that participants were 3-12 years old, of either sex, in good general health and with no known adverse reactions to permethrin-treated fabric, permethrin, or other insecticidal products. Independently witnessed, written informed consent for each child to participate was requested and received from the primary caregiver of all participants, after at least 24 hours to consider participation. Children aged 7-12 years also gave witnessed assent to participate. All participants were screened prior to testing, comprising a full health assessment by a nurse, and photos of the head and neck area were taken in case of skin complaints arising during the trial. A permethrin skin test was performed, in which participants were exposed to a small strip of the PTS for 72 hours to check for adverse reactions. For this, a section (approximately 25×4 cm) of intervention PTS was tied around the wrist. All personal information was anonymised using participant reference numbers.

All participants in all studies were free to withdraw at any time without giving a reason; no participant withdrew. The study protocol (available at https://doi.org/10.17037/DATA.00002423) was approved by the LSHTM ethics committee (LSHTM Ethics Ref: 15049‑01), the National Research Ethics Regulatory Committee Ethiopia/Ministry of Science and Higher Education and the Food, Medicine and Healthcare Administration and Control Authority of Ethiopia (02/25/33/06).

### Randomisation and masking

The 58 eligible children enrolled to the field trial were to receive either a PTS (intervention arm) or nothing (control arm) and were allocated to the two trial arms by the trial statistician using restricted randomization. The factors on which allocation was restricted were age, where the difference in mean age was restricted to be within 0.5 years in the two groups, and sex, where the difference in numbers of males between the two groups could not be more than one. A total of 100,000 possible permutations of allocations to arms were generated and those permutations that did not meet the restriction criteria were discarded. Of the remaining acceptable permutations, one was chosen using a computer-generated random number. Neither data collectors (entomological field workers) nor participants were masked to arm allocation.

### Procedures and outcomes

**Figure 3:**
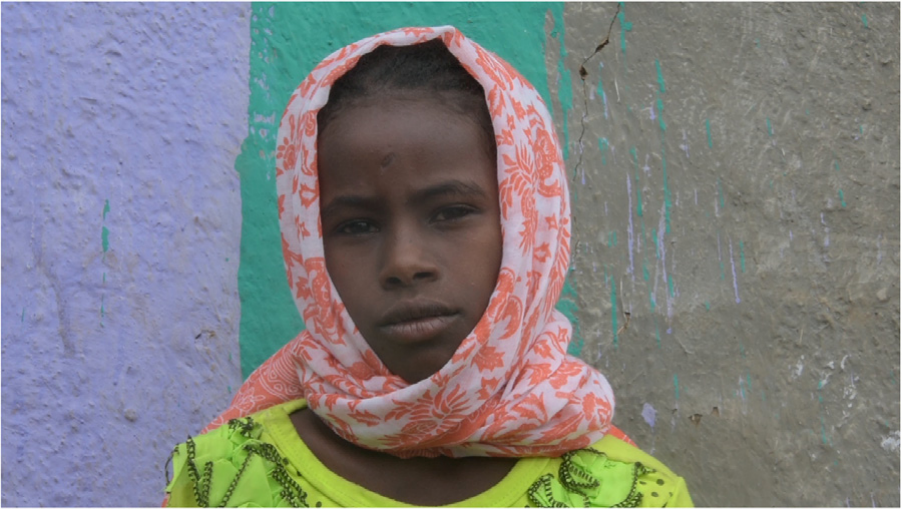
**A field study participant wearing the intervention product, a permethrin-treated headscarf**. These were Insect Shield® versatile wraps, 100% polyester and factory-treated with a proprietary permethrin formula using the “Insect Shield®” process, at a weight ratio of 0·52% w/w.

The preliminary laboratory trial tested Jungle Formula Kids, an over-the-counter insect repellent containing 15% active ingredient IR3535 (CAS Registry Number 52304-36-6), and hand-dipped permethrin-treated scarves (PTS; CAS 52645-53-1) (permethrin 0.017 and 0.034 mg/cm^2^, total amount 102 [PTS_102_] and 204 [PTS_204_] mg/scarf). These candidate products were tested for their protection against fly-skin contact using modified arm-in-cage bioassays to calculate the protective efficacy (supplementary materials, phase I studies: preliminary laboratory trial). The permethrin-treated scarves used as the field trial intervention were Insect Shield® versatile wraps (referred to as PTS) (Figure 3). These were 100% polyester scarves (91×183 cm) in coral with white print. Insect Shield® garments are factory-treated with a proprietary permethrin formula using the “Insect Shield®” process, at a weight ratio of 0·52% w/w. The PTS were manufactured in Zhejiang, China, and all PTS used in the field trial were from the same fabric/manufacturing lot. The permethrin content was verified by gas chromatography prior to dispatch. Scarves were worn as desired, but participants were encouraged to wear them close to or around the face. Generally, female participants chose to wear PTS as headscarves (Figure 3) while male participants chose to wear them around the neck. Participants in the control arm wore a placebo scarf (an identical scarf without Insect shield® treatment) for observation measures only, as distribution of placebo scarves for the period of intervention may have led to the mix-up of test and control items by participants.

The primary outcomes were fly-eye, -nose and -mouth contact, measured over several time points. On day 0 of the study, baseline measurement of fly-eye, -nose and -mouth contact was made. We collected data on several personal and environmental variables including ocular and nasal discharge (active discharge from the eye or nose, as distinct from simple eyelash crusting or crusting around the nose), participant biometrics (body weight, tympanic temperature) and environmental variables (ambient temperature (°C), light intensity, relative humidity (RH%), presence of rain or wind) as secondary outcomes. For baseline primary outcome measurement, all participants wore a placebo scarf while ten minutes of fly-face contact were recorded: participants sat on a chair, facing entomological field workers who tallied fly-eye, -nose and -mouth contacts from a distance of approximately 1.5 m. We defined (1) fly-eye contact as a fly touching the eye, lid edge or eyelashes; (2) fly-nose contact as a fly touching the nostril area; (3) fly-mouth contact as a fly touching the lips or lip margin. Repeat contacts by the same fly were recorded as new contacts, as differentiating between new and repeat contacts was not always possible. Observation periods were also filmed using a tripod-mounted camera (Nikon D7200 single-lens reflex). After baseline measurements, participants were given scarves (intervention arm: PTS, control arm: placebo scarf), then another ten minutes of fly-face contact was immediately recorded for all participants (time zero, T0). Fly-face contact measurements (10-min sessions) were then repeated at 30 minutes (T30), 1 hour (T60) and 3 hours (T180) later, with intervention arm participants continuing to wear their PTS from T0 onwards*.* The control arm participants were only asked to wear a placebo scarf during the 10-minute fly-face observation periods.

After the first day, participants in the intervention arm were encouraged to wear their PTS throughout each day for the 28-day duration of the study, which was governed by a compromise between maximising weeks of follow-up to measure continued efficacy and time constraints for study completion. Observations for all participants were repeated on day seven (D7) and day 28 (D28). On those follow-up days, fly-face contact measurements were done as per T0 measurements with both a control measurement (all participants wearing a placebo scarf) and a test measurement (intervention arm participants wearing their PTS; placebo arm participants wearing a placebo scarf). Secondary outcome measures were repeated on follow-up days. We encouraged adherence to the study (continued wearing of the PTS from day 0 to day 28 in intervention arm) at each visit by emphasizing that PTS could protect from trachoma and diarrhoeal disease transmission by flies. Between these visits, we made phone calls to a nominated local person who had a mobile phone, to encourage the participant's caregivers to support the child in adhering to protocol.

Study data were electronically captured in encrypted case report forms using tablets (Samsung Galaxy Tab E) programmed with the open-source survey tool kit ODK Collect. These were uploaded to a secure server as close to daily as possible. Protective efficacy (PE) against fly-face contact per participant was the planned effect measure, with PE calculated as the proportion of fly-face (fly-eye, -nose and -mouth) contact after application of the PTS (T) in relation to contacts before application of the PTS at baseline (C) (PE = 100*(1-(T/C))).^15^ As the field trial commenced it became apparent that this planned effect measure was inappropriate. Over the initial testing day (D0) the ambient temperature rose, causing fly contact counts to increase regardless of treatment allocation, leading to negative estimates of PE since the reference from which PE was calculated was taken early in the day. This was exacerbated on D7 and D28, which were further into the hot season. For analysis therefore, the effect measure used was the ratio of fly-eye, -nose and -mouth contacts between arms. As fly-eye contact is likely to be the most important outcome in terms of the transmission of trachoma, analysis and presentation of results focusses on this outcome. Adverse events were defined as any untoward medical occurrence in a study participant irrespective of the relationship with the intervention.

### Statistical analysis

We calculated that a sample size of 23 children per arm in the field trial would have 90% power to detect 30% difference in PE between the intervention arm and the control arm, assuming a standard deviation of 30%. We increased this to 29 participants per arm, 58 in total, to allow for 25% loss-to-follow-up. All enrolled participants were included in analyses unless absent.

The planned analysis was linear regression with individual measures of PE as the outcome and trial arm as the primary exposure; the results of this analysis are presented as supplementary information (supplementary materials, Table 1). Instead, we analyse and present the primary outcomes of fly-eye, -nose and -mouth contact as a between-arm comparison of the total number of contacts measured in the ten-minute observation periods. The effect of the intervention was estimated by a rate ratio obtained from a negative binomial regression, where number of fly contacts in ten minutes was the outcome and trial arm was the primary exposure. This was performed separately for each time point (T0, T30, T60, T180, D7. D28) and site (fly-eye, -nose and -mouth contact). A standard 5% significance level was used for these analyses despite multiple testing, as conventional multiple testing procedures would have been overly conservative because the outcomes are highly correlated.

**Table 1:** Baseline characteristics of the study populations. **PTS=permethrin-treated scarf. Data are n (%) or median (IQR).**

	Permethrin-treated scarf (n=29)	Control arm (n=29)
Sex		
Male	16 (55.2%)	16 (55.2%)
Female	13 (44.8%)	13 (44.8%)
Age (years)	6 (5-8)	6 (5-7)
Weight (kg)	20.7 (16.2-24.3)	18.4 (15.6-22)

Although there were no pre-specified factors for adjustment, results are presented as both unadjusted and adjusted for baseline (pre-intervention) contacts, as we observed a large imbalance in fly-face contacts at baseline. On follow-up days D7 and D28, control observations (10 minutes with placebo scarf) were performed for all participants as well as test observations (10 minutes with scarf according to intervention arm). We compared fly-face contact in these control observations using the same method.

To obtain an average estimate of the effect of trial arm on fly-eye, -nose and -mouth contact across all timepoints, a random-effects negative binomial regression model was used with fly (-eye, -nose and -mouth) contacts in ten minutes as the outcome, trial arm as the primary exposure and including timepoint as a categorical exposure. The same model was used to test whether the effectiveness of the scarf changed over time, also including an interaction term between day and trial arm. To assess the association of other personal and environmental exposures with the fly-face outcomes at baseline, negative binomial regression was used with exposure variables included one at a time. Associations were checked for potential confounding by adjusting for each of the unused variables one by one and the effect estimate checked for a substantial (∼10%) change in size. If multiple confounders were identified, then all were included in the model, however, because of the relatively small sample size we limited multiple adjustments to no more than four.

All statistical analyses were done in STATA version 15.1. The trial is registered with ClinicalTrials.gov (NCT03813069).

### Role of the funding source

The funder of the study had no role in study design, data collection, data analysis, data interpretation, or writing of the report. All authors had full access to all the data in the study and had final responsibility for the decision to submit for publication.

## Results

**Figure 4:**
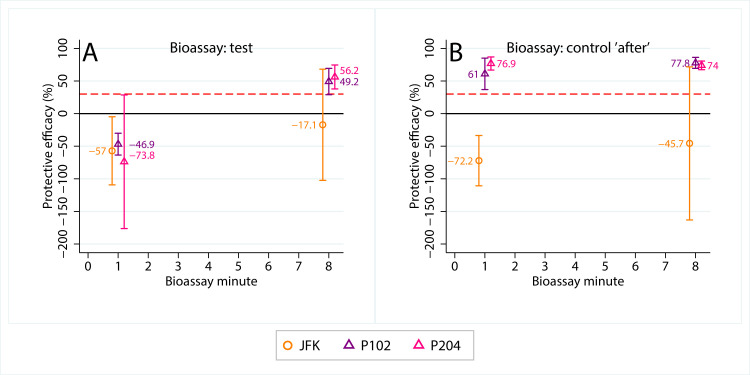
**Preliminary laboratory trial results**. Jungle Formula Kids® (JFK, orange markers) and permethrin-treated scarves (PTS; total amount permethrin 102 [P102, purple markers] and 204 [P204, pink markers] mg/scarf) were tested in a preliminary laboratory trial with six people. The product ‘test’ bioassays were run in-between control bioassays (‘before’ and ‘after’). Protective efficacy (PE) was calculated at each minute for both ‘test’ and control ‘after’ bioassays; PE was defined as the proportion of M. sorbens contacting the hand relative to that in the control ‘before’ bioassay. Points represent mean protective efficacy (+/- 95% confidence intervals), the red dashed line represents 30% PE, the threshold for use in the subsequent field trial.

Our repellent screening tests indicated that higher concentrations (15-20%) of topical repellents were effective against *M. sorbens* when applied to cover all exposed skin (supplementary materials). This would not be an appropriate intervention for protecting the peri-ocular area. Mixed levels of protection were observed for permethrin-treated clothing (supplementary materials). In the preliminary laboratory trial, PTS provided protection from fly contact that increased over time, from no observable protection in the first minute (PTS_102_: -46·9, 95% confidence interval [CI] -63·6 to -30·1; PTS_204_: -73·8, 95% CI -176·3 to 28·7), to good protection in the eighth minute (PTS_102_: 49·2, 95% CI 29·1 - 69·4; PTS_204_: 56·2, 95% CI 37·9 - 74·6) (Figure 4A). Surprisingly, protection was observed in the control (placebo scarf) bioassays run directly after test PTS bioassays (Figure 4B). This was not observed for JFK ‘control after’ bioassays, nor was any protection afforded in JFK test assays (Figure 4), despite the fact that JFK was intended as a positive control; this was in contrast to our earlier screening tests (supplementary materials, figure 4). Permethrin-treated scarves were, therefore, selected for field testing.

Sixty-seven participants consented and were recruited between Oct 29, 2019 and Nov 01, 2019 in the Kubi Guta kebele of Shashemene woreda, Oromia. Sixty-four were screened for eligibility, three found to be non-eligible, and the oldest three remaining children excluded to achieve the sample size (58). These participants, aged 4-10 years, were randomised to test or control arm (Figure 1, Table 1). The study was conducted between Nov 4, 2019 and Dec 23, 2019. All available participants were assessed for the primary endpoint. Four were excluded from D28 analyses (Figure 1). There were no adverse events or serious adverse events detected during the preliminary laboratory study nor the field trial.

**Figure 5:**
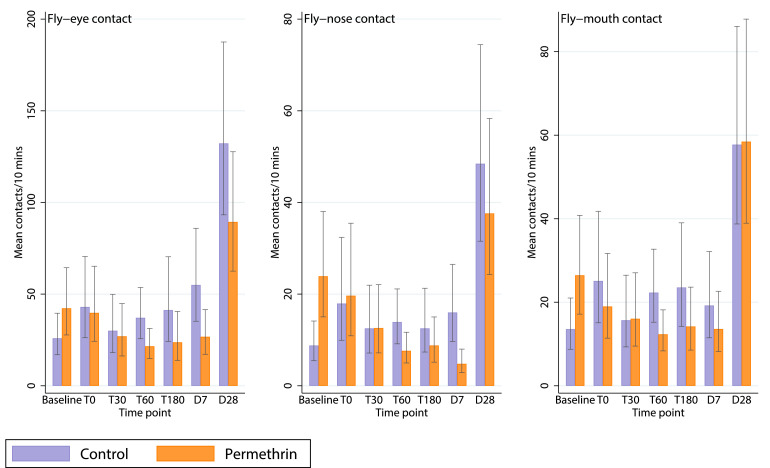
**Fly contacts experienced in the two study arms**. Number of fly-eye, -nose and -mouth contacts (mean in 10-minutes, predicted and unadjusted values; 95% confidence intervals) in intervention (permethrin, orange bars) and control (purple bars) arms (each n=29). Contacts were measured at baseline (before scarves were given), then at each time-point: immediately (T0), 30, 60 and 180 minutes later (T30/T60/T180), seven days later (D7) and 28 days later (D28).

We observed a greater number of fly-eye, -nose and -mouth contacts in the permethrin arm than in the control arm at baseline prior to any intervention (mean fly-eye contacts in ten minutes, control 25·90 [SD 26·22], permethrin 42·24 [SD 46·80]) (Figure 5, Table 2).

**Table 2:** Fly-eye contact observed in the intervention (permethrin) and control study arms. Raw data given and rate ratios of fly-eye contact in the permethrin arm relative to control, both adjusted and unadjusted for differences between arms at baseline.

	Control			Permethrin			RR permethrin vs control (95% CI) -Adjusted	*P*-value	RR permethrin vs control (95% CI) - Unadjusted	*P*-value
Timepoint	Mean contacts	SD	n	Mean contacts	SD	n				
Before intervention	25.9	26.22	29	42.24	46.8	29	NA	NA	NA	NA
Time zero (T0)	43	45.16	29	39.76	62.34	29	0.54 (0.29-0.98)	0.04	0.92 (0.46-1.86)	0.83
30 mins (T30)	30.03	32.49	29	26.97	36.98	29	0.60 (0.31-1.17)	0.13	0.90 (0.44-1.84)	0.77
60 mins (T60)	37.07	38.45	29	21.55	19.42	29	0.50 (0.31-0.79)	<0.01	0.55 (0.34-0.98)	0.04
180 mins (T180)	41.28	57.17	29	23.69	25.69	29	0.52 (0.25-1.07)	0.08	0.57 (0.27-1.22)	0.15
Day 7	54.96	92.52	27	26.71	25.39	28	0.41 (0.22-0.75)	<0.01	0.49 (0.26-0.91)	0.02
Day 28	132.22	115.96	27	89.35	81.1	26	0.62 (0.38-1.01)	0.05	0.68 (0.41-1.11)	0.12
Cross-timepoint	55.55	76.53	170	37.16	51.45	170	0.65 (0.52-0.83)	<0.01	0.81 (0.63-1.06)	0.12

SD=Standard Deviation, n=number of participants per arm, NA=Not Applicable.

After adjustment for the baseline value, evidence was found that children in the intervention arm immediately (at time 0, T0) experienced almost 50% fewer fly-eye contacts compared to children in the control arm (rate ratio [RR] 0·54, 95% confidence interval [CI] 0·29-0·98, *P*=0·04). Children in the intervention arm continued to wear their PTS throughout the first day of testing during which the adjusted results were fairly consistent: at follow-up timepoints T0, T30, T60 and T180, there were estimated reductions in fly-eye contacts of between 40 and 50% (Table 2). Protection in the intervention arm was more pronounced at D7 of the two follow-up days (D7 and D28), however, there was still some evidence of protection from fly-eye contact at D28 (D7: RR 0·41, 95% CI 0·22-0·75, *P*<0·001; D28: RR 0·62, 95% CI 0·38-1·01, *P*=0·05). Across all timepoints, we found a 35 % reduction in fly-eye contacts in the intervention arm relative to the control arm (RR 0·65, 95% CI 0·52-0·83, *P*<0·001), and we found no evidence that the effect of the intervention changed over time (*P*=0·38).

Without adjustment for baseline values of fly contact, there was no difference in fly-eye contact rates between arms for at least 30 minutes (Figure 5, Table 2). By 60 minutes a 45% reduction in fly-eye contact was observed in the intervention arm relative to the control arm (RR 0·55, 95% CI 0·34-0·98, *P*=0.04), and thereafter, estimated reductions of 30-50% (Table 2). Across all timepoints and without adjusting for baseline imbalance, there an approximately 20% reduction in fly-eye contacts in the intervention arm relative to the control arm (RR 0.81, 95% CI 0·63-1.06, *P*=0.12). Our analysis of protection against fly-eye contact as measured by protective efficacy provides some evidence of protection in the intervention arm at all timepoints (supplementary materials, Table 1).

Fly-nose and fly-mouth contact rates followed similar trends to fly-eye contact rates (Figure 5). After adjusting for the baseline value, there was an estimated 52% and 37% reduction in fly-nose and fly-mouth contact respectively at T0 in the intervention relative to control arm (RR 0·48, 95% CI 0·24-0·95, *P*=0·03; RR 0·63, 95% CI 0·31-1·29, *P*=0·21) (supplementary materials, Tables 2, 3). Thereafter, at all timepoints, fly contacts were lower in the intervention arm, with estimated reductions of between 28% and 83% (fly-nose) and 23 and 70% (fly-mouth). Across all timepoints, the adjusted analysis indicated a 31% and 21% reduction in fly-nose and fly-mouth contacts respectively in the intervention arm relative to the control arm (RR 0·69, 95% CI 0·51-0·92, *P*=0·01; RR 0·79, 95% CI 0·62-1.01, *P*=0.06). The unadjusted cross-timepoint analysis indicated a reduction of 24% of fly-nose contacts (RR 0·76, 95% CI 0·57-1.02, *P*=0·07), but no overall evidence of a reduction in fly-mouth contacts (RR 0·96, 95% CI 0·74-1.26, *P*=0.78).

**Table 3:** Fly-eye contacts observed using placebo scarves on follow-up days. On days D7 and D8, 10-minute control (placebo scarf) observations were made for all participants in addition to the 10-minute study arm measures (permethrin or control). Raw data given (mean/standard deviation [SD] and number of observations per arm [n]), and rate ratios of fly-eye contact in the permethrin arm relative to control, both adjusted and unadjusted for differences between arms at baseline.

Contacts in placebo observations	Control			Permethrin			RR permethrin vs control (95% CI) -Adjusted	*P*-value	RR permethrin vs control (95% CI) - Unadjusted	*P*-value
	Mean contacts	SD	n	Mean contacts	SD	n				
Day 7	33.85	56.09	27	13.68	22.28	28	0.38 (0.15-0.98)	0.05	0.4 (0.16-1.02)	0.06
Day 28	76.07	71.17	27	57.15	69.48	26	0.68 (0.35-1.32)	0.25	0.75 (0.39-1.46)	0.4

SD=Standard Deviation, n=number of participants per arm.

On follow-up days (D7 and D28), placebo scarf observations were made for participants in both arms. On D7, we found evidence that children in the intervention arm were afforded ‘residual’ protection from fly-eye contact (placebo observations; intervention arm vs. control arm: RR 0·38, 95% CI 0·15-0·98, *P*=0·05 [adjusted analysis]; RR 0·4, 95% CI 0·16-1.02, *P*=0·06 [unadjusted analysis]) (Table 3). There was less evidence of this residual protection from fly-eye contact at D28 (placebo observations; intervention arm vs. control arm: RR 0·68, 95% CI 0·35-1.32, *P*=0·25 [adjusted analysis]; RR 0·75, 95% CI 0·39-1.46, *P*=0·4 [unadjusted analysis]) (Table 3), and no evidence of this protection against fly-nose or fly-mouth contact (supplementary materials, Table 3). Approximately half of children in the intervention arm were recorded as wearing a PTS when the study team arrived on D7 and D28 (45% [n=13] and 55% [n=16], respectively).

Overall, flies were observed to visit eyes more frequently than the nose or mouth, with fly-eye contacts made approximately 1.8 and 2.8 times more frequently than fly-mouth and fly-nose contacts respectively. In all participants at baseline (prior to scarf distribution) we found no evidence that individuals with ocular discharge experienced more fly-eye, -nose or -mouth contact (supplementary materials, Table 4). However, we did find evidence that having nasal discharge increased the number of fly-nose contacts. After adjusting for body weight, we found evidence that fly-eye, -nose and -mouth contact was associated with participant age. Those in the youngest age group experienced the most contacts and this reduced with age (supplementary materials, Table 4). Of environmental variables measured, only ambient temperature at time of observation was found to influence fly contact rates, after adjusting for time of measurement of observations. For each °C increase in temperature, we saw a corresponding increase in fly contacts (23, 37 and 18% for fly-eye, -nose and -mouth, respectively) (supplementary materials, Table 4).

**Table 4:** Association between fly-eye contacts and other measured variables. Other person and environmental exposures were tested for their association with fly-eye contacts at baseline. Raw data given as well as rate ratios of fly contact relative to baseline.

			n	Mean contacts (SD)	Median contacts (IQR)	RR (95% CI)	P-value^A^	P-value^B^
Fly-eye contact								
Person variables	Ocular discharge	no	45	33.84 (40.17)	18 (4-40)	baseline		
		yes	13	34.85 (33.45)	29 (12-48)	1.03 (0.50-2.14)	0.94	
	Nasal discharge	no	32	32.34 (36.26)	18.5 (3.5-48.5)	baseline		
		yes	26	36.19 (41.70)	21 (11-43)	1.12 (0.61-2.06)	0.72	
	Age	4-5 yrs^C^	20	47.70 (48.78)	27 (12.5-72.5)	baseline		
		6 yrs	15	35.20 (34.49)	34 (4-50)	0.49 (0.21-1.18)^D^	0.11	
		7 yrs	10	32.10 (33.52)	22.5 (5-48)	0.46 (0.18-1.18)^D^	0.11	0.005
		8-10 yrs	13	13.31 (16.04)	6 (3-24)	0.14 (0.04-0.42)^D^	<0.001	
	Sex	Female	26	30.31 (37.26)	12.5 (3-40)	baseline		
		Male	32	37.13 (39.78)	31 (6.5-48.5)	1.22 (0.67-2.26)	0.52	
	Bodyweight, continuous					0.98 (0.91-1.05)	0.49	
	Tympanic temp, continuous					0.96 (0.59-1.55)^E^	0.86	
Environmental variables	Time of measurement	09:53-11:00	18	28.89 (33.79)	15.5 (3-50)	baseline		
		11:00-11:40	22	36.55 (37.49)	29 (6-43)	1.27 (0.61-2.64)	0.53	0.79
		11:40-12:50	18	36.22 (45.25)	19 (5-40)	1.25 (0.58-2.71)	0.57	
	Rel. humidity, continuous					1.01 (0.97-1.04)	0.69	
	Light intensity, continuous					1.0 (1.0-1.0)	0.75	
	Ambient temp, continuous					1.23 (1.02-1.50)^F^	0.03	

SD=Standard Deviation, n=number of participants per arm, IQR=Interquartile Range.

A P-value comparing this category with baseline.

B P-value testing hypothesis that variable is associated with number of fly contacts.

C Only five children aged four in participant group.

D Adjusted for bodyweight.

E Adjusted for participant age and ambient temperature.

F Adjusted for time of measurement (groups).

## Discussion

Our small-scale preliminary laboratory trial indicated that PTS, but not the topical repellent Jungle Formula Kids®, may reduce skin contact from *M. sorbens* flies. Based on this finding, we tested whether PTS could protect against eye-seeking *M. sorbens* flies in a phase II randomised field trial. From previous studies in this geographical area, we know that the eye-seeking fly population is around 90% *M. sorbens*.^14^ After adjusting for baseline values of fly contact, we found that PTS immediately halved fly-eye, -nose and -mouth contacts for the wearer, and that protection continued for the 28-day duration of the trial. These findings support our hypothesis that insect repellent products, but specifically ITC, may provide personal protection against *M. sorbens* fly-face contact.

We observed a large imbalance in fly (eye, nose and mouth) contacts in our baseline (pre-intervention) measures, with greater number of contacts in the PTS arm. Increased fly contacts are indicative of either higher fly density or increased host-seeking/aggression in the locality of the PTS arm children. Within 60 minutes, adjusted and unadjusted analyses show little difference between arms, indicating that either the source of imbalance was redressed, or the intervention effect was sufficient to overwhelm it. As such, adjusting for baseline values of fly contact has a large impact before 60 minutes, but little impact thereafter. While both analyses are given here, we present adjusted analyses as the primary results on the basis that it is correct to take the baseline imbalance into consideration.

Our field trial tested permethrin-treated scarves, treated with the pyrethroid insecticide permethrin. Permethrin is a neurotoxin, although it is important to note that synthetic pyrethroids are some of the least toxic-to-mammals insecticides in use,^16^ and exposure levels from ITC are very low.^17^ In insects, permethrin exposure can manifest as restlessness, incoordination, prostration and paralysis;^18^ we observed all of these in *M. sorbens* in our PTS laboratory bioassays. An insect's response to an insecticide is dependent on intrinsic and extrinsic factors and is dose-dependent, but can be broadly characterised in its action as toxic, contact irritant or spatial repellent.^19^ We observed in the laboratory that permethrin is a spatial repellent to *M. sorbens*, that is, these flies are stimulated to move away without making physical contact with the fabric. This mode of action allows the PTS to protect the user's skin even when not directly covered with fabric. In our laboratory studies, the action of permethrin developed over 8-minute test bioassays and continued to exert an effect for at least 8 minutes after the impregnated garment had been removed (control ‘after’ bioassays). This time-delay for the effect may explain the variable protection afforded by commercial ITC in screening tests (Appendix 1) in which fly contact was measured at minute 4-5. The ‘immediate’ effect of the PTS in the field study is consistent with this short time delay, as it took five to ten minutes to set up and prepare for the T0 measurement. Another study found that the full effect of permethrin on mosquitoes took several hours to develop.^20^ Negative protective efficacies in the preliminary laboratory trial are indicative of more fly contact in test/control ‘test’ bioassays, relative to the control ‘before’. This was probably because the eight-minute control ‘before’ bioassay had a stimulating effect in subsequent bioassays.

In the field trial intervention arm, across all timepoints we found a 35% decrease in fly-eye contact relative to the control arm. Insecticide space spraying was associated with a 96% and 88% reduction in fly-eye contact in two studies conducted in The Gambia,^5^^,^^6^ although fly-eye contact was not measured in precisely the same way. At our study site, fly density and clustering around the eyes was extreme; catching all flies to count them, as was undertaken in previous trials in the Gambia,^5^^,^^6^ was not possible here without disturbing the flies and altering the count. We therefore counted every contact as unique. This variation aside, achieving approximately 1/3 of the effect caused by space spraying is promising; modelling suggests that an intervention achieving a 10% reduction in Ct transmission intensity would have a marked programmatic impact in trachoma hyper-endemic populations.^21^ The implementation of an ITC fly control programme may prove to be simpler, cheaper, and more acceptable than spraying residual insecticide at scale.

To the best of our knowledge, to date this is the first trial of the use of any repellent product against African *M. sorbens*. Insect repellents have been used successfully against the Australian bush fly *M. vetustissima* (Walker) (Muscidae),^22^ which is in the three-species *M. sorbens* complex.^23^ Aerosol formulations of a permethrin transfluthrin mix, sprayed onto substrates at a high application rate (60 g of 0.1/0.05% permethrin), have also been found to afford protection from fly landings (population approximately 80% *M. domestica* (Linneus) (Muscidae) and 20% *M. vetustissima*) in an area 1 metre from treated surfaces.^24^ Several studies demonstrate successful use of repellent products against the only blood-seeking filth fly, *Stomoxys calcitrans* (Linneus) (Muscidae).^25^, ^26^, ^27^

Despite preparatory phase I studies, modifications were made to the study protocol for data analysis after fieldwork had commenced. Protocol modifications were made appropriately, in response to evolving knowledge of how permethrin affected the flies and seasonal changes in the field setting (i.e. increased fly density as the study progressed into the hot season). It is important to note that while the effect measure for the primary outcomes, protective efficacy, was replaced with analysis of the rate ratio, this alteration did not change the underlying parameters and assumptions of the study design. Both effect measures compare fly contact rates between arms. The performance of multiple tests (three primary outcome measures at six timepoints) could be considered a further limitation of the analysis, therefore *P* values should be taken in the context of multiple testing. However, fly-eye, -nose and -mouth contacts on an individual are highly correlated outcomes (governed by a host's individual and intrinsic attractiveness to these flies) and so cannot be considered independent outcomes. We therefore report the *P* values as calculated rather than adjusting for multiple testing. Finally, fly contacts were measured by trained entomological fieldworkers, and while this is an accepted method of measuring nuisance from eye-seeking flies,^6^ the lack of masking means that the study is potentially subject to observer bias. While it is conceivable that observer bias led to the baseline imbalance in fly counts, it seems unlikely given that all participants wore placebo scarves during baseline measures, and subconcious inflation of intervention arm participant fly count does not seem intuitive.

It will be important to replicate and measure the longevity of PTS repellency, ideally in a large-scale phase III study. Insect shield® items are factory treated with permethrin such that the repellency should last 70 washes. While the assocation between trachoma prevalance and water scarcity may reduce the number of washes garments experience, high levels of UV light in trachoma-endemic environments may contribute to rapid degradation of permethrin. It might be useful to also trial other permethrin-treated items, for example hand-dipped scarves, which could be cheaper (but might have reduced longevity of repellency). We found evidence that on D7 of the trial children in the intervention arm still benefitted from fewer fly-eye contacts in the absence of the PTS. This is supportive of the possibility that residual permethrin on the skin continued to repel flies,^28^ as seen in the preliminary laboratory trial, and this effect should be studied in more depth. Further, this finding coroborates our observations that PTS were well-received by the study participants, who continued to wear these garments throughout the study period. Acceptability studies concerning the intervention items will the subject of a separate manuscript. We recorded that approximately half of children in the intervention arm were wearing a PTS when the study team arrived on D7 and D28, however, the real value may be different as these data were not consistently recorded. Finally, future studies should also address whether PTS reduce attack by other disease vectors.

The phase II studies described here show that PTS can protect against eye-seeking flies. Larger, phase III trials with epidemiological outcomes are warranted to corroborate and extend these findings. Permethrin-treated clothing could be an effective weapon against the transmission of trachoma.

## Contributors

AR, JGL, AL and MJB conceived the study, AR and JGL designed the study, AR, LROG, OSA, WA, GS, EM, MG, DL, EE, KT, KHK, SML, SLW, EH, DA, FS and MAA implemented the study, AR and DM verified the underlying data, carried out the statistical analysis and interpreted the results, AR did the literature search and wrote the first draft of the manuscript, JGL, MJB, AL and AWS supervised the study, VS, JGL and MJB obtained funding for the study. All authors reviewed and approved the final manuscript before submission.

## Declaration of interests

The authors have nothing to disclose.

## Data sharing statement

De-identified individual participant data on which statistical analysis including summary figures and tables are based, an accompanying data dictionary defining each field, and related documents including the study protocol, participant information sheets and consent forms will be made available from the point of, and for 10 years after, the acceptance for publication of the main findings from the final dataset. Datasets will be publicly available at LSHTM Data Compass (https://doi.org/10.17037/DATA.00002423).

## Appendix Supplementary materials

Image, application 1

Image, application 2

Image, application 3
