# Supplementary material for: Evaluation of the efficacy of insecticide-treated scarves to protect children from the trachoma vector Musca sorbens (Diptera: Muscidae): A phase II randomised controlled trial in Oromia, Ethiopia
Source: eClinicalMedicine. 2022 Jun 8;49:101487. doi: 10.1016/j.eclinm.2022.101487 (PMC9189873; doi:10.1016/j.eclinm.2022.101487)
Supplement: Supplementary file 1 [file mmc1.docx]

**SUPPLEMENTARY MATERIAL**

## Phase 1 studies: bioassay design

Figure 1. **Flies of different physiological status (sex or diet status) were assayed for aggressiveness (skin-seeking behaviour) using modified arm-in-cage methods**. Numbers represent paired bioassays; in the sex assays pairs represent 50 males and 50 females from the same egg batch, in the diet assays pairs represent 50 flies with access to protein and 50 without from the same egg batch.

## Phase 1 studies: repellent screening

Figure 2. **Screening insect repellent products against *M. sorbens*** (A) A 6cm-diameter circle of repellent was applied to the top of the hand, and fly behaviour was analysed within a 2-cm margin around the hand. The circle of repellent approximated the volume of repellent considered safe to apply to a child’s cheek, the margin of fly observation approximated the distance required to protect the eye. White and pink lines represent one-centimetre bands, calibrated using a section of ruler included in the frame. (B) Biotracking software (Bio-tracking, Georgia Tech) was used to determine minutes of fly-skin contact time for the targeted image area.

Figure 3. **Phase 1 studies screened various insect repellent products for possible efficacy against *M. sorbens***. Protective efficacy here is the proportion of cumulative fly contact in one minute of the test assay (minute 4) relative to the average cumulative fly contact (in minute 4) in the controls run before and afterwards. Spatial experiments (E-G) reflect the use of a small volume of topical repellent applied to the top of the hand, and fly contact measured a 2 cm-area around the hand. Insecticide-treated clothing (ITC) tested was Craghoppers shirt [CH shirt], Insect shield scarf [IS scarf and Insect shield shirt [IS shirt], all were tested wrapped around the wrist and fly contact on the hand measured.

**Phase 2 results**

**Protective Efficacy**

Table 1. **Results by Protective Efficacy**. The phase 2 study was designed to have a primary outcome of protective efficacy against fly contacts, calculated per individual as the proportion of fly-face (fly-eye, -nose and -mouth) contact after application of the PTS (T) in relation to contacts before application of the PTS at baseline (C) (PE = 100*(1-(T/C))). Mean/standard deviation (SD) PE against fly-eye contact per arm, and coefficients from linear regression, are shown. Samples sizes per timepoint per arm deviate from those presented in between-arm comparison because the PE calculation does not allow for zero contacts at baseline.

**Fly-nose and fly-mouth contacts**

Table 2. **Fly-nose and -mouth contacts observed in the intervention (permethrin) and control study arms.** Raw data given and rate ratios of fly-eye contact in the permethrin arm relative to control, both adjusted and unadjusted for differences between arms at baseline.

Table 3. **Fly-nose and -mouth contacts observed using placebo scarves on follow-up days**. On days D7 and D8, 10-minute control (placebo scarf) observations were made for all participants in addition to the 10-minute study arm measures (permethrin or control). Raw data given (mean/standard deviation [SD] and number of observations per arm [n]), and rate ratios of fly-eye contact in the permethrin arm relative to control, both adjusted and unadjusted for differences between arms at baseline.

Table 4. **Other person and environmental exposures were tested for their association with fly-nose and -mouth contacts at baseline**. Raw data given as well as rate ratios of fly contact relative to baseline.
