# Supplementary material for: Evaluation of the efficacy of insecticide-treated scarves to protect children from the trachoma vector Musca sorbens (Diptera: Muscidae): A phase II randomised controlled trial in Oromia, Ethiopia
Source: eClinicalMedicine. 2022 Jun 8;49:101487. doi: 10.1016/j.eclinm.2022.101487 (PMC9189873; doi:10.1016/j.eclinm.2022.101487)
Supplement: Supplementary file 2 [file mmc2.docx]

**SUPPLEMENTARY MATERIAL**

## Phase 1 studies: bioassay design

### Methods

Experiments aimed to identify the physiological status of the most aggressively skin-seeking *M. sorbens* for use in modified arm-in-cage bioassays. One investigator conducted all assays, who avoided the use of fragranced cosmetics or washing products for 12 hours prior to testing and did not use tobacco. Immediately before testing, the participant’s arm was washed with Simple odour-free soap (Unilever, UK), rinsed with water, rinsed with 70% ethanol (Sigma-Aldrich, UK), then towel dried. For each bioassay repetition, the forearm was inserted into the cage (Bugdorm, Taiwan) containing 50 flies. Fly behaviour on and around the arm/hand was filmed for 10 minutes using a camera (Canon G12) whose lens pointed down through a hole in the top of the cage. Bioassay design and all other phase I studies described here were conducted in testing facilities at the London School of Hygiene & Tropical Medicine in London. The testing room temperature was maintained between 19-29°C and 15-90% RH, and all tests were conducted in the diel phase between 09:00 and 17:00.

To test the effect of fly sex on aggression (skin contact), males and females from the same egg batch were compared in separate assays on the same day. This was repeated six times. Flies were 4-10 days old (post-eclosion) and had acclimatised in the testing room without access to water or sugar for 12 hours prior to testing. The testing room was maintained at 26·7-27·5°C and 21-27% humidity. To test the effect of fly diet on aggression, flies (25 male/25 female) who had been given access to a protein source from eclosion (organic whole milk powder, Buy Whole Foods Online Ltd, UK) were compared against 50 flies (25 male/25 female) from the same egg batch who had received only sugar and water. Flies were compared in separate assays on the same day, this was repeated four times. Flies were 7-9 days post-eclosion and had acclimatised in the testing room without access to water or sugar for 12 hours prior to testing. *Musca sorbens* (﻿Wiedemann) (narrow frons form) were reared in the LSHTM in-house insectary according to published methods (Robinson 2021, in press).

1000 frames of video footage were analysed for total fly-skin contact time. Prior to tracking, a background image was masked to focus tracking on and around the hand/arm. Software (Bio-tracking, Georgia Tech) was then used to determine minutes of fly-skin contact time for the targeted timeframe and image area. Fly-skin minutes were summed and differences in paired bioassays determined by paired t-tests.

### Results

Female flies spent more time on the skin than male flies (*P*=0.05). Although flies who were deprived a protein source spent more time on the skin, the observed difference did not reach statistical significance (*P*=0.13) (Figure 1).

Figure 1. **Flies of different physiological status (sex or diet status) were assayed for aggressiveness (skin-seeking behaviour) using modified arm-in-cage methods**. Numbers represent paired bioassays; in the sex assays pairs represent 50 males and 50 females from the same egg batch, in the diet assays pairs represent 50 flies with access to protein and 50 without from the same egg batch.

## Phase 1 studies: repellent screening

### Methods

One investigator screened a range of insect repellent products for usefulness against *M. sorbens*. Each repellent/repellent product was tested once per day for three days. Testing times (per day) were kept uniform, and order of testing randomised by Latin Square. Per experiment, a specific cage was allocated to each repellent/repellent product, which was washed in warm soapy water and dried thoroughly prior to use. Female *M. sorbens* (3+ days old [post-eclosion]) were starved of a protein source from eclosion, with access to only sugar and water, and were kept with males up until that point to allow mating to occur. Flies were taken to testing area at least 12 hours prior to testing to acclimatize, with access to sugar and water only. All bioassays were run for eight minutes. Further details per experiment are given below.

#### (1) Dose response (DEET, IR3535, Picaridin, dUDL)

Analytical standards of *N*,*N*-diethyl-3-methylbenzamide (DEET), insect repellent 3535 (ethyl 3-[acetyl(butyl)amino]propanoate; IR3535) and delta-Undecalactone (6-hexyloxan-2-one; dUDL) were obtained from ﻿Sigma-Aldrich, UK, picaridin (butan-2-yl 2-(2-hydroxyethyl)piperidine-1-carboxylate) was obtained from Bertin Pharma, France, commercially available Smidge® (20% picaridin) was obtained directly from Smidge (APS Biocontrol Ltd, Dundee, UK). All topical products were applied at the standard laboratory application rate of 1 ml product/600 cm^2^. Dose response experiments tested standards at five incrementally increasing doses up to a maximum of 20% (DEET/IR35353/ dUDL, 1/5/10/15/20%; picaridin, 1/4/7/10/13%), and all were diluted in ethanol (Sigma-Aldrich, UK). Prior to repellent testing, diluent alone was tested as a control (‘before’ control). One ml ethanol was applied to the arm and allowed to dry (1 min), then the arm was inserted into the cage. For the test to proceed there had to be 5+ fly contacts in the first eight-minute observation period. After this, the lowest dose of repellent in ethanol (1 ml) was applied and allowed to dry (1 min). The arm was re-inserted and insect behaviour filmed for eight minutes. This was repeated for each incremental dose, all were applied serially on top of the previous application, up to a maximum of 20% active ingredient. After all doses had been tested, 1 ml of the diluent control was applied to the investigator’s other arm and tested again (‘after’ control), as per the ‘before’ control, to verify continued fly contact/landings.

#### (2) Spatial effect (topical IR3535, Picaridin, dUDL)

To be useful in the field, any topical repellent would have to be applied in small volume on the cheek and protect the eye region with a spatially repellent effect. Therefore, the volume of repellent appropriate for a 6 cm diameter circle (28 cm^2^) was applied to the back of the hand, representing the volume of repellent that could be applied to a child’s cheek. Analysis focussed on a 2 cm-margin around the hand (Figure 2). Assays were otherwise conducted as in (1), with two doses (10/20%) of topical IR3535, Picaridin and dUDL being tested serially, in-between controls (ethanol alone). DEET was excluded as a candidate at this stage because of safety concerns around using it close to the eyes.

**(A)**


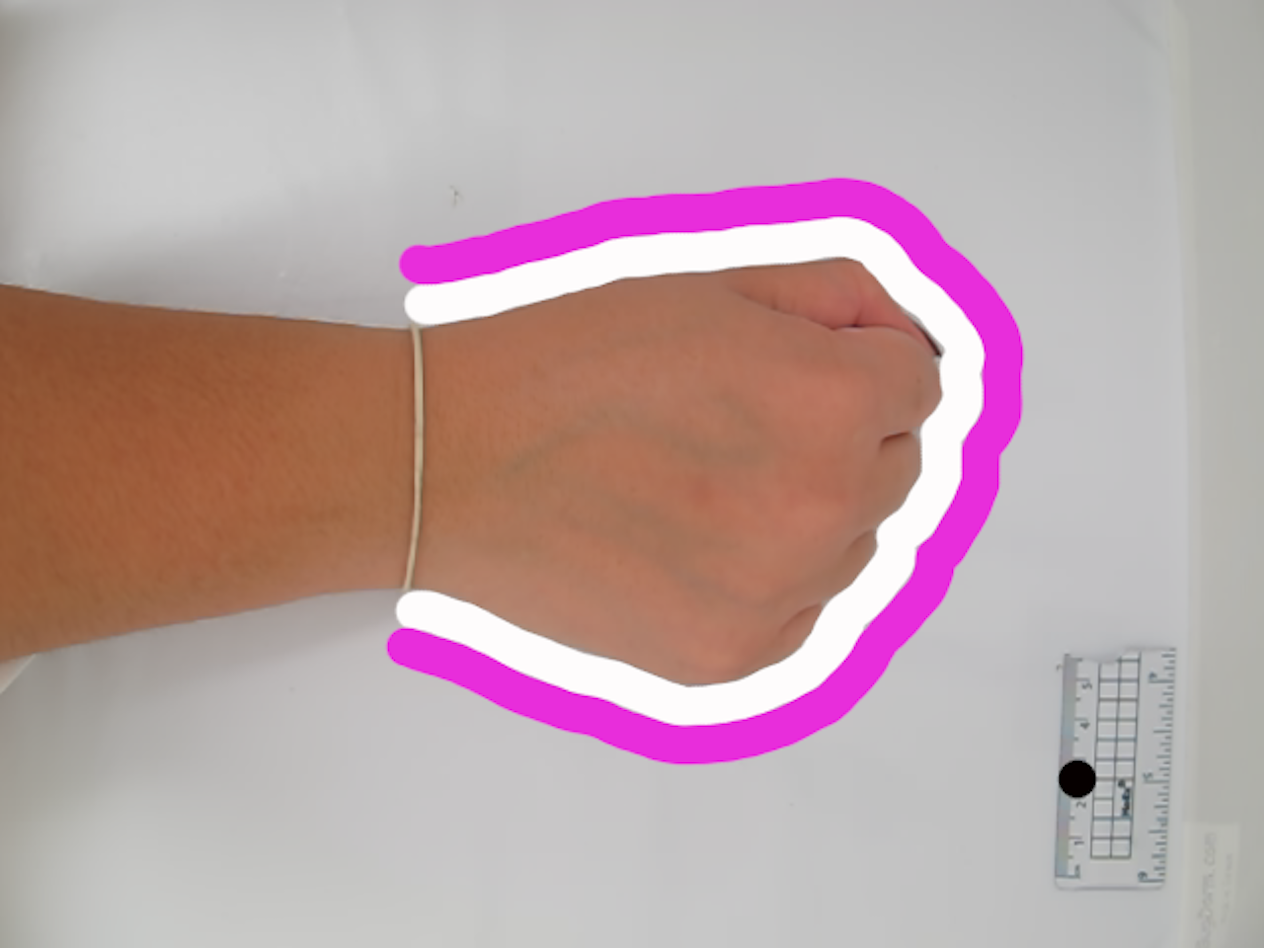


**(B)**


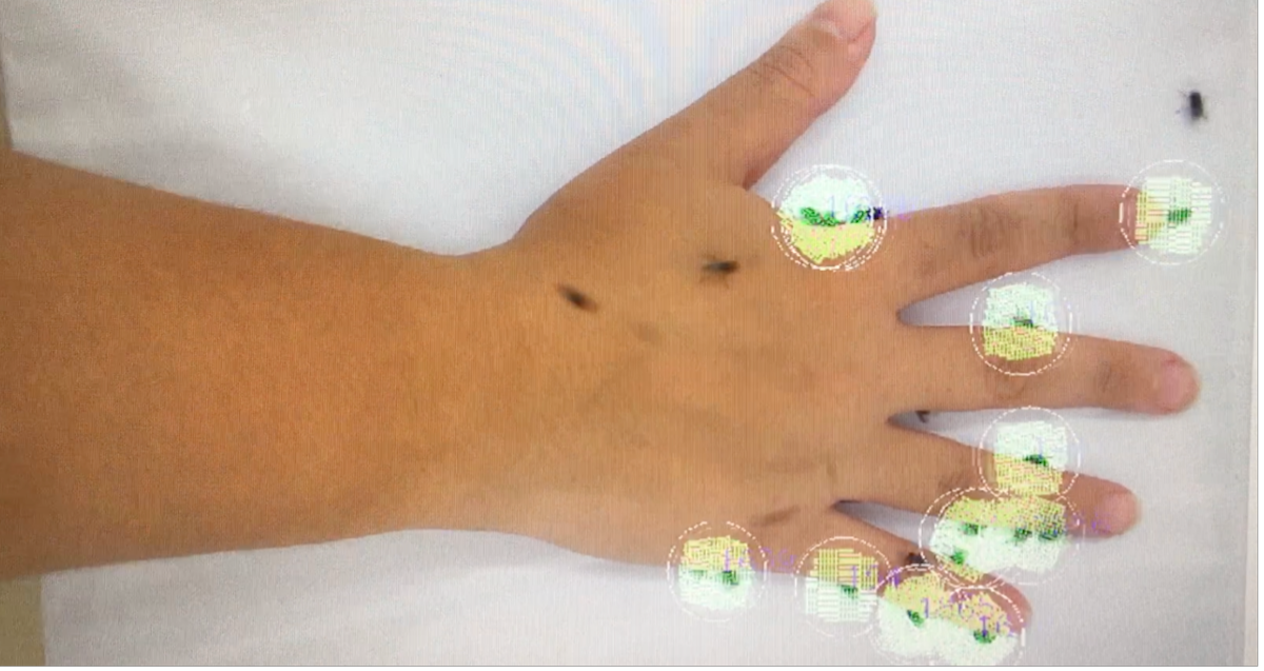


Figure 2. **Screening insect repellent products against M. sorbens** (A) A 6cm-diameter circle of repellent was applied to the top of the hand, and fly behaviour was analysed within a 2-cm margin around the hand. The circle of repellent approximated the volume of repellent considered safe to apply to a child’s cheek, the margin of fly observation approximated the distance required to protect the eye. White and pink lines represent one-centimetre bands, calibrated using a section of ruler included in the frame. (B) Biotracking software (Bio-tracking, Georgia Tech) was used to determine minutes of fly-skin contact time for the targeted image area.

#### (3) Insecticide treated clothing (Craghoppers/Insect shield)

Insecticide-treated clothing (ITC) containing the active ingredient (a.i.) permethrin was tested with a view to using headwear or scarves in the field trial. Garments were worn around the wrist and fly contact on the hand measured, therefore, assays tested for a spatial effect. Bioassays were conducted as in (1), with no product used in control assays (bare arms). Insect shield® clothing (shirt/scarf) is treated with Insect shield formulation containing 0.52% permethrin, Craghopper® clothing (shirt) is treated with 0.50% w/w permethrin﻿. For shirts, test sleeves were cut near the shoulder seam and the loose sleeves will used as a normal sleeve, secured with two elastic bands. The scarf was wrapped around the wrist and secured with an elastic band.

For experiments 1-3, minute 4-5 of video footage was analysed for total fly-skin contact time per bioassay. Prior to tracking, a background image was masked to focus tracking on and around the hand/arm, masking criteria were uniform per experiment. Software (Bio-tracking, Georgia Tech) was then used to determine minutes of fly-skin contact time for the targeted image area (Figure 2B). Fly-skin minutes were summed and protective efficacy (PE) calculated as a proportion of the cumulative fly-skin contact time on the treated arm (T) in relation to the mean cumulative fly-skin contact time on the control arm measured before and after (C) (*PE=100*(1-(T/C))*)^1^. For all screening assays statistical comparison was not made due to small sample sizes (n=3).

### Results and discussion

The topical repellents DEET, IR3535, picaridin and dUDL, when used according to manufacturer’s instructions and applied as a complete coat over the hand and arm, were observed to reduce fly contact and give good PE at the higher concentrations tested in dose-response experiments (experiment 1; 63·9, 75·9, 98·9% PE for 20% DEET; 98·6, 96·0, 100% for 20% IR3535; 98·7, 95·4, 91·5% for 13% picaridin; 78·7, 100, 100, 100% for 20% dUDL, Figure 3A, B, C, D). However, little protection from fly contact was observed for any topical repellent in the ‘spatial’ experiments (experiment 2, Figure 3E, F, G). The protection afforded by ITC was variable (Craghopper shirt; 0, 0, 39.5%, Insect shield scarf 5, 37.9, 100%, Insect shield shirt 33.5, 53.7, 76.2%; Figure 3H). In these screening tests, good protection was observed from 15-20% topical IR3535 when used over the hand and arm at the standard laboratory application rate. Topical IR3535 was, therefore, used in the subsequent preliminary laboratory trial as a positive control, alongside the best field intervention candidate, permethrin-treated scarves.


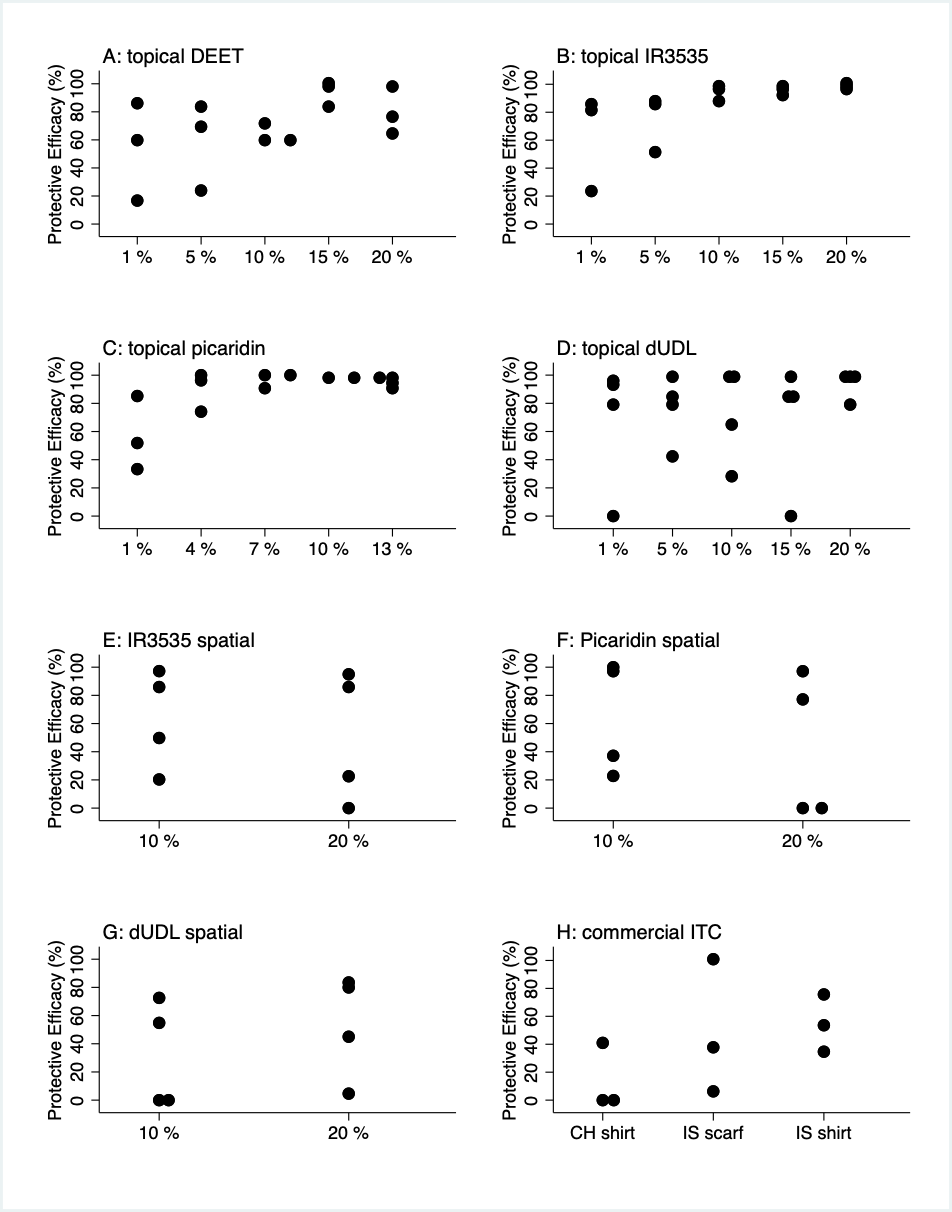


Figure 3. **Phase 1 studies screened various insect repellent products for possible efficacy against M. sorbens**. Protective efficacy here is the proportion of cumulative fly contact in one minute of the test assay (minute 4) relative to the average cumulative fly contact (in minute 4) in the controls run before and afterwards. Spatial experiments (E-G) reflect the use of a small volume of topical repellent applied to the top of the hand, and fly contact measured a 2 cm-area around the hand. Insecticide-treated clothing (ITC) tested was Craghoppers shirt [CH shirt], Insect shield scarf [IS scarf and Insect shield shirt [IS shirt], all were tested wrapped around the wrist and fly contact on the hand measured.

## Phase 1 studies: preliminary laboratory trial

### Study design and participants

We conducted a small within-subject, non-randomised laboratory trial of the use of the topical repellent Jungle Formula Kids (JFK), and permethrin-treated scarves (PTS), against contact from *M. sorbens*. Each of six participants tested all three investigational products (IP) three times over three days, with the order of testing per day randomised by Latin square design. Eligible participants were 18-65 years old, both sexes, in full health and with no known adverse reactions to the commercially available repellents DEET (*N*,*N*-diethyl-3-methylbenzamide), PMD (para-Menthane-3,8-diol), IR3535 (ethyl 3-[acetyl(butyl)amino]propanoate), Picaridin (butan-2-yl 2-(2-hydroxyethyl)piperidine-1-carboxylate) or Permethrin (3-phenoxyphenyl)methyl 3-(2,2-dichloroethenyl)-2,2-dimethylcyclopropane-1-carboxylate). Independently witnessed, written informed consent was requested and received from all participants, and ﻿all were screened prior to testing for adverse reactions to the IPs by low-dose exposure, followed by 72 hours of monitoring.

### Procedures and outcomes

Jungle Formula Kids (London, UK) was commercially sourced and applied to the hand and arm at the standard laboratory application rate of 1 mL product/600 cm^2^. PTS were made from grey cotton fabric (100x60 cm, John Lewis, London). Scarves were first washed in warm water with detergent (﻿Teepol; Merck, Dorset, United Kingdom), rinsed in hot water, rinsed in 70% EtOH then Millipore-filtered water, and dried in drying cabinets. Analytical grade permethrin (Yorlab, York, UK) was solubilised in hexane (Scientific Laboratory Supplies Limited, Nottingham, UK) at two concentrations of 0.017 and 0.034 mg/cm^2^ (total amount 102 [PTS_102_] and 204 [PTS_204_] mg/scarf) and the PTS immersed in these solutions. Concentrations were designed not to exceed the AEL_medium term_ (Acceptable exposure level for medium term use; 0.05 mg/kg bw/day)^2^ for a child weighing 10 kg, assuming 0.49% dermal absorption.^3^ Control PTS were immersed in hexane only. Once the solutions were completely absorbed, PTS were dried in a drying cabinet and stored in foil at room temperature until use. For bioassays, PTS were folded, wrapped around the wrist and secured with an elastic band. Investigational products were tested using 8-minute modified arm-in-cage bioassays (described above); a control bioassay was run before and after each ‘test’ (IP) assay. Data collectors and participants were not blinded to which intervention was being tested. In JFK control assays no product (bare arm) was used. During testing, room temperature was maintained at 19·9-23·7°C and RH 32-61%. Bioassays were filmed to retrospectively quantify fly-skin contact, the primary outcome. Participant and bioassay data were electronically captured in encrypted case report forms using a tablet (Samsung Galaxy Tab E) programmed with the open-source survey tool kit ODK Collect. These were uploaded onto encrypted external hard drives daily.

To quantify fly-skin contact, we counted the total number of flies touching the arm on each minute of the 8-minute assay. Flies were counted if any part of the fly was in contact with the arm at that time, data collectors (counting flies) were blinded as the appearance of control/test assays was identical per IP. Protective efficacy against fly-skin contact was the primary outcome. In the protocol, PE was the proportion of fly-skin contact on the treated arm in the test assay (T) in relation to fly-skin contact on the arm in the control assays run before and after the test assay (C) (*PE = 100*(1-(T/C)*)).^1^ We deviated from this definition of PE at analysis because there was a clear time-lag for permethrin to take effect in PTS test assays, and because the flies were still profoundly affected by permethrin in the control run afterwards. (C) was therefore redefined as fly-skin contact on the arm in the control assay run before only. Per protocol assessment of the persistence of repellency was not conducted due to time constraints.

### Statistical analysis

We estimated the mean protective efficacy (and associated 95% confidence interval) at minutes 1 and 8 during the ‘test’ bioassay (after introduction of the IP) and during the ‘control after’ bioassay. For IP to qualify for testing in the subsequent field trial a PE of at least 30% against fly contact was required. All participants were included in statistical analysis of the primary outcome. A data monitoring committee reviewed these preliminary trial results prior to the field trial commencing.

**Phase 2 results**

**Protective Efficacy**

Table 1. **Results by Protective Efficacy**. The phase 2 study was designed to have a primary outcome of protective efficacy against fly contacts, calculated per individual as the proportion of fly-face (fly-eye, -nose and -mouth) contact after application of the PTS (T) in relation to contacts before application of the PTS at baseline (C) (PE = 100*(1-(T/C))). Mean/standard deviation (SD) PE against fly-eye contact per arm, and coefficients from linear regression, are shown. Samples sizes per timepoint per arm deviate from those presented in between-arm comparison because the PE calculation does not allow for zero contacts at baseline.

| Timepoint | Control | | | Permethrin | | | Coefficient, permethrin vs control (95% CI) | *P*-value |
| --- | --- | --- | --- | --- | --- | --- | --- | --- |
|  | Mean PE | SD | n | Mean PE | SD | n |  |  |
| T0 | -181.35 | 371.72 | 27 | -11.56 | 102.29 | 28 | 169.79 (23.50-316.08) | 0.02 |
| T30 | -122.00 | 420.66 | 27 | 13.92 | 91.15 | 28 | 135.92 (-27.31-299.16) | 0.10 |
| T60 | -132.83 | 379.50 | 27 | -37.38 | 175.76 | 28 | 95.46 (-63.55-254.47) | 0.23 |
| T180 | -142.90 | 357.69 | 27 | -46.13 | 225.04 | 28 | 96.77 (-64.23-257.77) | 0.23 |
| D7 | -385.35 | 710.04 | 27 | -30.70 | 154.36 | 28 | 354.66 (73.48-635.84) | 0.01 |
| D28 | -1018.56 | 1378.40 | 27 | -792.71 | 2020.05 | 28 | 225.86 (-743.91-1195.62) | 0.64* |

*D28 result is influenced by one large outlier in the permethrin arm, *P* value for the non-parametric test=0.04

**Fly-nose and fly-mouth contacts**

Table 2. **Fly-nose and -mouth contacts observed in the intervention (permethrin) and control study arms.** Raw data given and rate ratios of fly-eye contact in the permethrin arm relative to control, both adjusted and unadjusted for differences between arms at baseline.

|  | Control | | | Permethrin | | | RR permethrin vs control (95% CI) -Adjusted | *P*-value | RR permethrin vs control (95% CI) - Unadjusted | *P*-value |
| --- | --- | --- | --- | --- | --- | --- | --- | --- | --- | --- |
|  | Mean contacts | SD | n | Mean contacts | SD | n |  |  |  |  |
| Fly-nose contact |  |  |  |  |  |  |  |  |  |  |
| Before intervention | 8.79 | 11.98 | 29 | 23.90 | 36.44 | 29 | NA | NA | NA | NA |
| Time zero (T0) | 17.93 | 29.95 | 29 | 19.66 | 37.50 | 29 | 0.48 (0.24-0.95) | 0.03 | 1.10 (0.48-2.53) | 0.83 |
| 30 mins (T30) | 12.52 | 18.68 | 29 | 12.59 | 25.53 | 29 | 0.41 (0.21-0.82) | 0.01 | 1.01 (0.45-2.23) | 0.99 |
| 60 mins (T60) | 13.90 | 15.01 | 29 | 7.62 | 8.52 | 29 | 0.34 (0.20-0.57) | <0.001 | 0.55 (0.30-1.00) | 0.05 |
| 180 mins (T180) | 12.52 | 18.87 | 29 | 8.79 | 11.55 | 29 | 0.50 (0.24-1.02) | 0.057 | 0.70 (0.33-1.49) | 0.36 |
| Day 7 | 16.00 | 19.87 | 27 | 4.79 | 7.86 | 28 | 0.17 (0.08-0.38) | <0.001 | 0.30 (0.15-0.61) | <0.01 |
| Day 28 | 48.44 | 78.27 | 27 | 37.62 | 39.71 | 26 | 0.72 (0.41-1.29) | 0.28 | 0.78 (0.42-1.43) | 0.42 |
| Cross-timepoint | 19.94 | 38.26 | 170 | 14.84 | 27.11 | 170 | 0.69 (0.51-0.92) | 0.01 | 0.76 (0.57-1.02) | 0.07 |
| Fly-mouth contact |  |  |  |  |  |  |  |  |  |  |
| Before intervention | 13.55 | 20.04 | 29 | 26.45 | 30.50 | 29 | NA | NA | NA | NA |
| Time zero (T0) | 25.10 | 31.27 | 29 | 19.00 | 20.93 | 29 | 0.63 (0.31-1.29) | 0.21 | 0.76 (0.37-1.56) | 0.45 |
| 30 mins (T30) | 15.69 | 20.00 | 29 | 16.03 | 17.21 | 29 | 0.77 (0.39-1.54) | 0.46 | 1.02 (0.49-2.14) | 0.95 |
| 60 mins (T60) | 22.31 | 22.17 | 29 | 12.31 | 10.40 | 29 | 0.48 (0.29-0.80) | <0.01 | 0.55 (0.32-0.95) | 0.03 |
| 180 mins (T180) | 23.55 | 30.25 | 29 | 14.21 | 14.21 | 29 | 0.50 (0.25-1.01) | 0.06 | 0.60 (0.29-1.23) | 0.17 |
| Day 7 | 19.22 | 22.60 | 27 | 13.61 | 27.38 | 28 | 0.30 (0.14-0.65) | <0.01 | 0.71 (0.34-1.46) | 0.35 |
| Day 28 | 57.74 | 53.89 | 27 | 58.46 | 71.69 | 26 | 0.80 (0.44-1.43) | 0.45 | 1.01 (0.57-1.79) | 0.97 |
| Cross-timepoint | 27.01 | 34.29 | 170 | 21.68 | 36.10 | 170 | 0.79 (0.62-1.01) | 0.06 | 0.96 (0.74-1.26) | 0.78 |

SD=Standard Deviation, n=number of participants per arm, NA=Not Applicable.

Table 3. **Fly-nose and -mouth contacts observed using placebo scarves on follow-up days**. On days D7 and D8, 10-minute control (placebo scarf) observations were made for all participants in addition to the 10-minute study arm measures (permethrin or control). Raw data given (mean/standard deviation [SD] and number of observations per arm [n]), and rate ratios of fly-eye contact in the permethrin arm relative to control, both adjusted and unadjusted for differences between arms at baseline.

|  | Control | | | Permethrin | | | RR permethrin vs control (95% CI) -Adjusted | *P*-value | RR permethrin vs control (95% CI) - Unadjusted | *P*-value |
| --- | --- | --- | --- | --- | --- | --- | --- | --- | --- | --- |
|  | Mean contacts | SD | n | Mean contacts | SD | n |  |  |  |  |
| D7: Fly contact in placebo observations | | |  |  |  |  |  |  |  |  |
| Fly-nose | 11.07 | 20.9 | 27 | 11.68 | 24.4 | 28 | 0.44 (0.16-1.18) | 0.1 | 1.05 (0.44-2.55) | 0.91 |
| Fly-mouth | 13.52 | 15.84 | 27 | 16.68 | 28.91 | 28 | 0.69 (0.25-1.92) | 0.11 | 1.23 (0.53-2.90) | 0.63 |
| D28: Fly contact in placebo observations | | |  |  |  |  |  |  |  |  |
| Fly-nose | 35.04 | 48.48 | 27 | 27.92 | 33.38 | 26 | 0.70 (0.34-1.45) | 0.34 | 0.80 (0.38-1.69) | 0.56 |
| Fly-mouth | 32.3 | 34.45 | 27 | 46.08 | 53.52 | 26 | 1.20 (0.59-2.44) | 0.61 | 1.39 (0.68-2.83) | 0.37 |

SD=Standard Deviation, n=number of participants per arm.

Table 4. **Other person and environmental exposures were tested for their association with fly-nose and -mouth contacts at baseline**. Raw data given as well as rate ratios of fly contact relative to baseline.

|  |  |  | n | Mean contacts (SD) | Median contacts (IQR) | RR (95% CI) | P-value^A^ | P-value^B^ |
| --- | --- | --- | --- | --- | --- | --- | --- | --- |
| Fly-nose contact |  |  |  |  |  |  |  |  |
| Person variables | Ocular discharge | no | 45 | 16.67 (30.79) | 4 (2-18) | baseline |  |  |
|  |  | yes | 13 | 15.23 (15.21) | 7 (6-28) | 0.91 (0.39-2.12) | 0.83 |  |
|  | Nasal discharge | no | 32 | 8.44 (11.60) | 4 (1-10.5) | baseline |  |  |
|  |  | yes | 26 | 26.08 (37.88) | 10 (4-39) | 2.31 (1.11-4.84)^G^ | 0.03 |  |
|  | Age | 4-5^C^ yrs | 20 | 26.95 (41.07) | 8 (4.5-41) | baseline |  |  |
|  |  | 6 yrs | 15 | 16.47 (21.72) | 5 (1-28) | 0.36 (0.12-1.13)^D^ | 0.08 |  |
|  |  | 7 yrs | 10 | 8.90 (10.42) | 4.5 (2-12) | 0.19 (0.06-0.67)^D^ | 0.01 | 0.006 |
|  |  | 8-10 yrs | 13 | 5.62 (6.86) | 3 (1-6) | 0.10 (0.03-0.40)^D^ | 0.001 |  |
|  | Sex | Female | 26 | 9.38 (12.25) | 4 (1-11) | baseline |  |  |
|  |  | Male | 32 | 22.00 (35.22) | 7 (2.5-33.5) | 1.75 (0.89-3.44)^H^ | 0.1 |  |
|  | Bodyweight, continuous |  |  |  |  | 0.94 (0.87-1.02) | 0.14 |  |
|  | Tympanic temp, continuous |  |  |  |  | 0.88 (0.49-1.56)^E^ | 0.65 |  |
| Environmental variables | Time of measurement | 09:53-11:00 | 18 | 21.44 (41.55) | 4.5 (2-28) | baseline |  |  |
|  |  | 11:00-11:40 | 22 | 15.91 (19.48) | 8 (3-20) | 0.74 (0.32-1.71) | 0.48 | 0.41 |
|  |  | 11:40-12:50 | 18 | 11.78 (19.31) | 5 (1-12) | 0.55 (0.23-1.32) | 0.18 |  |
|  | Rel. humidity, continuous |  |  |  |  | 1.03 (0.98-1.07) | 0.21 |  |
|  | Light intensity, continuous |  |  |  |  | 1.0 (1.0-1.0) | 0.08 |  |
|  | Ambient temp, continuous |  |  |  |  | 1.37 (1.1-1.7)^F^ | 0.004 |  |
| Fly-mouth contact |  |  |  |  |  |  |  |  |
| Person variables | Ocular discharge | no | 45 | 21.04 (29.29) | 9 (2-25) | baseline |  |  |
|  |  | yes | 13 | 16.38 (11.98) | 17 (8-26) | 0.78 (0.36-1.67) | 0.52 |  |
|  | Nasal discharge | no | 32 | 15.22 (19.47) | 7 (2-24.5) | baseline |  |  |
|  |  | yes | 26 | 25.88 (32.44) | 17 (4-27) | 1.42 (0.69-2.94)^G^ | 0.34 |  |
|  | Age | 4-5^C^ yrs | 20 | 32.05 (34.73) | 25 (6.5-38.5) | baseline |  |  |
|  |  | 6 yrs | 15 | 18.40 (26.32) | 7 (1-25) | 0.28 (0.11-0.75)^D^ | 0.01 |  |
|  |  | 7 yrs | 10 | 11.60 (9.57) | 11 (4-17) | 0.18 (0.06-0.52)^D^ | 0.001 | 0.001 |
|  |  | 8-10 yrs | 13 | 9.77 (10.21) | 6 (1-17) | 0.12 (0.04-0.36)^D^ | <0.001 |  |
|  | Sex | Female | 26 | 14.04 (20.10) | 6.5 (2-21) | baseline |  |  |
|  |  | Male | 32 | 24.84 (30.01) | 17 (5-31) | 1.56 (0.81-2.99)^H^ | 0.19 |  |
|  | Bodyweight, continuous |  |  |  |  | 0.97 (0.91-1.04) | 0.45 |  |
|  | Tympanic temp, continuous |  |  |  |  | 1.16 (0.71-1.89)^E^ | 0.56 |  |
| Environmental variables | Time of measurement | 09:53-11:00 | 18 | 17.39 (27.52) | 6.5 (2-17) | baseline |  |  |
|  |  | 11:00-11:40 | 22 | 20.68 (21.83) | 19 (4-31) | 1.19 (0.55-2.56) | 0.66 | 0.85 |
|  |  | 11:40-12:50 | 18 | 21.78 (31.27) | 11.5 (4-27) | 1.25 (0.56-2.80) | 0.58 |  |
|  | Rel. humidity, continuous |  |  |  |  | 0.99 (0.96-1.03) | 0.65 |  |
|  | Light intensity, continuous |  |  |  |  | 1.0 (1.0-1.0) | 0.14 |  |
|  | Ambient temp, continuous |  |  |  |  | 1.18 (0.99-1.41)^F^ | 0.06 |  |

SD=Standard Deviation, n=number of participants per arm, IQR=Interquartile Range

^A^ P-value comparing this category with baseline

^B^ P-value testing hypothesis that variable is associated with number of fly contacts

^C^ Only five children aged four in participant group

^D^ Adjusted for bodyweight

^E^ Adjusted for participant age and ambient temperature

^F^ Adjusted for time of measurement (groups)

^G^ Adjusted for presence of ocular discharge, participant age and sex

^H^ Adjusted for nasal discharge

**References**

1 World Health Organization. Guidelines for efficacy testing of mosquito repellents for human skin. WHO Pesticide Evaluation Scheme (WHOPES); 2009.

2 European Commission. Assessment Report: Permethrin. 2014.

3 Snodgrass HL. Permethrin transfer from treated cloth to the skin surface: Potential for exposure in humans. J Toxicol Environ Health. 1992; **35**(2): 91–105.
